# Outcomes of stenting with overlapping drug-eluting stents versus overlapping drug-eluting and bare-metal stents for the treatment of diffuse coronary lesions

**DOI:** 10.5830/CVJA-2010-004

**Published:** 2010-12

**Authors:** SE Kassaian, M Salarifar, M Raissi Dehkordi, M Alidoosti, E Nematipour, HR Poorhosseini, AM Hajizeinali, D Kazemisaleh, A Sharafi, M Mahmoodian, N Paydari, AV Farahani

**Affiliations:** Department of Interventional Cardiology, Tehran Heart Center, Tehran University of Medical Sciences, Tehran, Iran; Department of Interventional Cardiology, Tehran Heart Center, Tehran University of Medical Sciences, Tehran, Iran; Department of Interventional Cardiology, Tehran Heart Center, Tehran University of Medical Sciences, Tehran, Iran; Department of Interventional Cardiology, Tehran Heart Center, Tehran University of Medical Sciences, Tehran, Iran; Department of Interventional Cardiology, Tehran Heart Center, Tehran University of Medical Sciences, Tehran, Iran; Department of Interventional Cardiology, Tehran Heart Center, Tehran University of Medical Sciences, Tehran, Iran; Department of Interventional Cardiology, Tehran Heart Center, Tehran University of Medical Sciences, Tehran, Iran; Department of Interventional Cardiology, Tehran Heart Center, Tehran University of Medical Sciences, Tehran, Iran; Department of Interventional Cardiology, Tehran Heart Center, Tehran University of Medical Sciences, Tehran, Iran; Department of Interventional Cardiology, Tehran Heart Center, Tehran University of Medical Sciences, Tehran, Iran; Department of Interventional Cardiology, Tehran Heart Center, Tehran University of Medical Sciences, Tehran, Iran; Department of Interventional Cardiology, Tehran Heart Center, Tehran University of Medical Sciences, Tehran, Iran

**Keywords:** angioplasty, stents, restenosis, overlapping stents, drug-eluting stents

## Abstract

**Introduction:**

We investigated the outcomes of stenting with overlapping drug-eluting stents (DES) versus overlapping stenting with a combination of drug-eluting and bare metal stents (BMS) in very long ≥(≥ 25 mm).

**Methods and Results:**

Fifty-two patients treated with either overlapping DES-DES (*n* = 22) or DES-BMS (*n* = 30) were selected from a registry of 588 patients with very long coronary lesions. Patients with acute myocardial infarction (MI) within the preceding 48 hours were excluded. The DES-DES combination was more frequently used for longer lesions compared with the DES-BMS group (47.95 ± 9.25 vs 39.98 ± 9.15 mm, *p* = 0.003). Left anterior descending artery lesions were also more frequently treated with the DES-DES combination (95.5 vs 66.7%, *p* = 0.02). In four patients in the DES-BMS group, overlapping stents were used for the coverage of dissections. Peri-procedural non-Q-wave MI occurred in one patient in the DES-BMS group. On follow up, only one case of non-fatal MI occurred in a patient with overlapping DES-DES.

**Conclusion:**

Overlapping a BMS in the proximal part of a long DES instead of exclusive deployment of two or more overlapped DES seems to be a safe and feasible therapeutic strategy in our practice.

## Summary

Percutaneous intervention in long ≥was previously associated with poorer short- and long-term results than that in discrete lesions.^1^ Owing to their associated low angiographic and procedural success rates and their higher dissection and threatened or acute vessel closure rates, these lesions were considered a contraindication to balloon angioplasty.^2^ Nevertheless, promising results were obtained when using drug-eluting stents (DES), making these lesions inviting targets for percutaneous coronary intervention (PCI).^3^-^5^

In the bare-metal stent (BMS) era, there were conflicting data regarding the efficacy of overlapping stents for the treatment of long lesions.^1^,^6^-^8^ However, in the contemporary DES era, multiple stent implantations in long lesions have been widely used.^4^,^5^,^9^ Nevertheless, concerns remain about potential local toxicity with delayed endothelial coverage that could contribute to an excess in very late stent thrombosis, most likely at adjacent DES strut overlaps,^10^ and also greater in-stent late lumen loss and angiographic restenosis.^11^

In our practice, combinations of drug-eluting and bare-metal stents have sometimes been used for covering very long lesions. One of the reasons was the unavailability of appropriate sizes of DES for overlapping coverage of these types of lesions, especially during the first two years of DES usage. Another reason was the high cost of DES in our country, which many people could not afford. However, the use of an additional stent was occasionally unplanned in cases such as dissections. To our knowledge, few clinical studies have to date systematically investigated the outcomes of a combination of overlapping DES and BMS.

In this study at our centre, we report on cases of multiple overlapping stenting in two groups of patients with implantation of DES and a combination of DES and BMS in ≥of ≥ 25 mm in length.

## Methods

DES were first used at our centre in March 2003 in selected, planned procedures. Then, on the basis of contemporary studies, their use increased so that by March 2004, DES were used as often as BMS. Since then, the use of DES at our institute has steadily increased, accounting for about 60% of the procedures in the last year.

Between April 2003 and March 2005, a total of 588 consecutive patients with lesions ≥ 25 mm underwent PCI at the catheter laboratory of our hospital, which performs 2 000 PCI procedures per year using six expert operators. After the exclusion of patients with acute MI within the preceding 48 hours of the procedure, a total of 52 patients were diagnosed for multiple overlapping stent implantation in their coronary arteries, using at least two overlapping stents, including one or more DES.

All angioplasty procedures were done with a 6 or 7 French guiding catheter and a femoral approach. If judged possible by the operator, direct stenting was attempted; however, in most cases pre-dilatation with an undersized balloon was used before DES implantation. High-pressure (> 12 atm) inflation was used in the majority of cases (*n* = 40, 76.9%). Stent overlaps were always post-dilatated at higher pressure with a non-compliant balloon or with the balloon of a proximally implanted stent. In total, 114 stents were used for the coverage of 52 lesions.

Seventy-eight drug-eluting stents were used: 63 sirolimuseluting stents (Cypher, Johnson & Johnson Cordis Corp) and 15 paclitaxel-eluting stents (Boston Scientific Corp, Natick, MA). The bare-metal stents used were diverse: one S670, one S660 and two S7 (Medtronic AVE, Minneapolis, MN), seven Biodivysio (Biocompatibles, Galway, Ireland), seven BX-Sonic (Cordis/Johnson & Johnson, Warren, MI), six Driver (Medtronic Vascular, Santa Rosa, California), six Express 2 (Boston Scientific, Natick, MA), three Vision, and three Zeta (Guidant, Santa Clara, CA).

Baseline, clinical, angiographic and procedural characteristics and in-hospital outcomes were obtained by research physicians and entered into a computerised database by computer operators. Finally, clinical outcomes, including major adverse cardiac events (MACE) were obtained by research physicians in clinics at one, six and 12 months post surgery, and once a year thereafter, or by formal telephone interviews, and these were recorded in datasheets, which were later entered into our computerised database.

All patients gave individual written informed consent for participation in this study and for the PCI procedure. The Ethics Committee in our centre approved this study, according to the Declaration of Helsinki as revised in 2000.

## Coronary procedures

Patients received 300 mg of clopidogrel and 325 mg of aspirin before and 7 500–10 000 IU of heparin at the start of the procedure. The femoral sheath was removed after normalisation (< 40 s) of the partial thromboplastin time. Clopidogrel was followed at a dose of 75 mg/day for at least three months in 2003, and at least six months since 2004, and aspirin was given indefinitely to all patients. Beta-blockers, angiotensin converting enzyme inhibitors and statin drugs were administered as appropriate in the absence of a specific contraindication. Abciximab was not used in any cases. Angiographic findings such as vessel dimensions, pre- and post-procedural stenoses, lesion length, and thrombolysis in myocardial infarction (TIMI) flow grade were determined by visual estimation.

## Definitions

Angina symptoms were defined according to the classification of the Canadian Cardiovascular Society.^12^ Lesion types were noted according to the American College of Cardiology/American Heart Association (ACC/AHA) lesion characteristics classification.^13^ Q-wave MI was defined as the presence of new Q waves in post-procedure electrocardiogram, with a two-fold increase in MB fraction of creatinine kinase. Non-Q-wave MI was defined as a two-fold increase in MB fraction of creatinine kinase without the development of new Q waves.

Angiographic success was defined as residual stenosis < 20% plus normal TIMI flow grade 3. Procedural success was defined as angiographic success without major complications (death, MI, emergency bypass surgery or PCI) during hospitalisation. MACE was defined as the presence of cardiac death, non-fatal MI, or target-vessel revascularisation (TVR) during the follow-up period. TVR was defined as ischaemia-driven repeat percutaneous intervention or bypass surgery of the target vessel. Target lesion revascularisation (TLR) was defined as ischaemia-driven repeat percutaneous intervention of the target lesion or bypass surgery of the target vessel.

## Statistical analysis

Numerical variables were presented as mean ± SD, while categorised variables were shown as numbers (%). Continuous variables were compared using the Student’s *t*-test or non-parametric Mann-Whitney *U*-test whenever the data did not appear to have normal distributions, and categorical variables were compared using the Chi-square or Fischer’s exact test. The analyses were conducted with SPSS software version 13 for Windows (Statistical Package for the Social Sciences, Inc, Chicago, IL). All *p*-values were two-tailed, with statistical significance taken as *p* < 0.05.

## Results

## Baseline and immediate results

During the study period, 22 patients were treated with two homogenous overlapping DES (in 17 cases, two overlapping Cypher stents; and in five, two overlapping Taxus stents were used). Thirty patients were treated with at least one DES combined with at least one BMS in an overlapping manner (in 25 cases, Cypher stents and in five cases, Taxus stents were used, combined with a variety of bare-metal stents, as stated in the methods section). In 23 cases, only one BMS was used. In four cases, two BMS, and in three cases, three bare-metal stents were used for the coverage of residual lesions. All the overlapping stents were placed for very long lesions (mean lesion length in the total population: 43.36 ± 9.93 mm).

The patients’ clinical characteristics are listed in Tables 1 and 2. As shown, the patients in these two groups were similar in baseline demographic characteristics. Lesions were estimated to be on average longer and the mean inflation pressure per lesion was higher in the DES-DES group, compared with the DES-BMS group (47.95 ± 9.25 vs 39.98 ± 9.15 mm, *p* = 0.003, and 16.29 ± 2.26 vs 14.52 ± 2.22 atm, *p* = 0.01, respectively). Moreover, a higher percentage of procedures were performed on the left anterior descending artery in the DES-DES group (Tables 2, 3).

**Table 1.** Selected Baseline Clinical Characteristics In Patients Treated With Overlapping Drug-Eluting Stents Versus Combined Bare-Metal And Drug-Eluting Stents

	*DES-DES (n = 22) n (%)*	*DES-BMS (n = 30) n (%)*	p*-value*
Female gender	4 (18.2)	8 (26.7)	0.53
Positive family history	7 (31.8)	15 (50)	0.19
Age (years)	56.13 ± 10.75	54.80 ± 14.63	0.71
Smoking	14 (63.6)	14 (46.7)	0.22
Diabetes mellitus	2 (9.1)	9 (30)	0.09
Hyperlipidaemia	13 (59.1)	19 (63.3)	0.76
Hypertension	5 (22.7)	11 (36.7)	0.28
MI history	8 (36.4)	12 (40)	0.79
Prior PCI	1 (4.5)	0	0.42
Prior CABG	0	3 (10)	0.25

*Mean ± SD. MI: myocardial infarction; PCI: percutaneous coronary intervention; CABG: coronary artery bypass grafting.

**Table 2.** Lesion Characteristics In Patients Treated With Overlapping Drug-Eluting Stents Versus Combined Bare-Metal And Drug-Eluting Stents

	*DES-DES (n = 22) n (%)*	*DES-BMS (n = 30) n (%)*	p*-value*
Type B2/C lesions	24 (100)	30 (100)	–
Multi-vessel disease	7 (31.8)	12 (40)	0.56
Pre-procedural stenosis* (%)	90.95 ± 9.08	92.07 ± 6.50	0.61
Lesion length*	47.95 ± 9.25	39.98 ± 9.15	0.003
Ostial lesion	1 (4.5)	0	0.42
Lest anterior descending artery	21 (95.5)	20 (66.7)	0.02
Right coronary artery	1 (4.5)	8 (26.7)	0.06
Left circumflex artery	0	2 (6.7)	0.50
Calcified	2 (9.1)	4 (13.3)	> 0.999
Eccentric	3 (13.6)	7 (23.3)	0.49
Angulated segments	1 (4.5)	6 (20)	0.22
Thrombus	0	1 (3.3)	> 0.999
Total occlusion	5 (22.7)	3 (10)	0.26

*Mean ± SD.

**Table 3.** Procedural Characteristics In Patients Treated With Overlapping Drug-Eluting Stents Versus Combined Bare-Metal And Drug-Eluting Stents

	*DES-DES (n = 22) (Cypher-Cypher: 17) (Taxus-Taxus: 5) n (%)*	*DES-BMS (n = 30) (Cypher-BMS: 25) (Taxus-BMS: 5) n (%)*	p*-value*
Stent length (mm)*^#^	50.91 ± 8.35	55.79 ± 16.37	0.17
Mean stent length per lesion* (mm)	25.68 ± 4.34	23.81 ± 3.96	0.11
Mean stent diameter per lesion* (mm)	2.86 ± 0.25	2.94 ± 0.35	0.34
Mean stent inflation pressure per lesion* (atm)	16.29 ± 2.26	14.52 ± 2.22	0.01
Number of stents per lesion			
2	22 (100)	23 (76.7)
3	0	4 (13.3)
4	0	3 (10)

*Mean ± SD, ^#^the reported stented length is based on the cumulative length of the adjacent stents.

During hospitalisation, angiographic failure occurred in one patient in each group. In the DES-BMS group, non-Q-wave MI occurred in one patient (Table 4). He was treated with two overlapping Cypher stents (2.75 × 33 mm) and one Express 2 (3.5 × 8 mm). Six months later, he underwent repeat coronary angiography, which showed patent stents.

**Table 4.** In-Hospital And Late Clinical Outcomes In Patients Treated With Overlapping Drug-Eluting Stents Versus Combined Bare-Metal And Drug-Eluting Stents

	*DES-DES (n = 22) n (%)*	*DES-BMS (n = 30) n (%)*	p*-value*
*In-hospital outcomes*			
Peri-procedural non-Q-wave MI	0	1 (3.3)	> 0.999
Angiographic success	21 (95.5)	29 (96.7)	> 0.999
Procedural success	21 (95.5)	28 (93.3)	> 0.999
Dissection	0	4 (13.3)	0.13
Abrupt closure	0	0	–
*Long-term outcomes*	*n = 22 (100%)*	*n = 28 (93.3%)*	
Mean follow-up duration (months)	13.9 ± 4.2	13.3 ± 4.8	0.65
MACE	1 (4.5)	0	0.44
Cardiac death	0	0	-
Non-fatal MI	1 (4.5)	0	0.44
TVR	0	0	-
TLR	0	0	-
CABG	0	0	-

*Mean ± SD. MI: myocardial infarction; MACE: major adverse cardiac events; TVR: target vessel revascularisation; TLR: target lesion revascularisation; CABG: coronary artery bypass grafting.

All dissections were treated with a combination of drug-eluting and bare-metal stents (Table 4). In two cases, four stents; and in one case, three stents were used; in one patient, two stents were used for the coverage of lesions. In all cases, the first implanted stent was a DES. In the case with three stents, the second stent was also a DES. All the other stents were BMS. In our total population, the mean length and diameter of DES vs BMS was 27.96 ± 6.53 vs 16.53 ± 7.59 mm (*p* < 0.0001) and 2.58 ± 0.32 vs 3.01 ± 0.43 mm (*p* = 0.025), respectively. In the DES-BMS group, the mean length and diameter of DES vs BMS was 31.21 ± 3.67 vs 16.53 ± 7.59 mm (*p* < 0.001) and 2.84 ± 0.35 vs 3.01 ± 0.43 mm (*p* = 0.071), respectively.

## Late outcomes

The mean follow-up duration was 13.5 ± 4.6 months. Of the 52 patients, 50 were followed up with clinic visits: all 22 patients treated with overlapping DES and 28 of the 30 patients treated with a combination of DES and BMS. At 30 days’ and six months’ follow up, MACE did not occur in any patient.

MI did however occur in a patient treated with two overlapping Cypher stents (3 × 33 and 3 × 18 mm). He was an ex-smoker and the diseased location was distal LAD. He continued to receive clopidogrel for seven months. After 13 months, he was admitted due to post MI angina. Re-angiography showed minimal CAD and development of coronary aneurysm in the coronary segment that had received overlapping stents. The patient underwent implantation of an intra-cardiac defibrillator (ICD) due to ventricular fibrillation and ejection fraction of 15%.

With univariate analysis, the composite endpoint of TVR, MI and cardiac death (MACE) was not related to any variable listed in Table 4.

## Discussion

In the present report, we describe the clinical outcomes of a small, consecutive series of patients treated with overlapping DES or overlapping DES and BMS. The data presented here demonstrate that overlapping stents may be used for the treatment of long coronary lesions, with both a high acute success rate and a good mid-term clinical outcome.

Long ≥comprise up to 20% of current interventional practice and are considered difficult both technically and in terms of achieving successful clinical outcomes.^1^,^14^ To date, both single long stents and multiple contiguous stents have been used for the successful treatment of coronary artery lesions.^5^,^15^-^17^

Stent length was previously an independent predictor of restenosis; however, the use of DES has greatly attenuated this relationship. As a result, long DES tend to be selected for complete lesion coverage, but if this approach is not possible or a residual segment of the lesion is left uncovered, additional stenting is considered, with some overlap to eliminate the risk of a residual stent gap.^18^,^19^ Some reports have shown an increased rate of peri-procedural myonecrosis in overlapping stents, which may be a limitation of this approach.^9^,^20^ A pooled analysis of five clinical trials showed that BMS overlap was associated with an increased incidence of MI and total MACE that was not apparent for DES overlap.^11^

On the other hand, Finn *et al.* reported that, in a histological analysis in an animal study, overlapping DES further delayed the arterial healing and promoted inflammation compared to overlapping BMS.^21^ Therefore they concluded that patients receiving overlapping DES needed more frequent follow up than patients with non-overlapping stents. It is noteworthy that many studies show comparable results after PCI with homogeneous and heterogeneous drug-eluting stents,^22^,^23^ and a low rate of repeat revascularisation irrespective of stent type, with no safety concerns at medium-term follow up.^24^,^25^

In 2006, Burzotta *et al.* distributed their report on a consecutive series of 40 patients in Italy, treated with overlapping stents. These stents were used to cover minor dissections and plaque shifts, treat other contiguous lesions in the same vessel, or obtain full lesion coverage. In a subgroup of patients, an appropriate (length and size) similar type of DES was not available, so the additional stent necessary to complete the procedure was a BMS or another type of DES. In their study, 24 patients were treated with overlapping homogeneous DES, eight with overlapping heterogeneous DES, and eight with overlapping DES-BMS. In their experience, three out of the 24 patients (12.5%) in the overlapping homogeneous DES group developed MACE: two, target lesion revascularisations and one, coronary bypass surgery. No MACE occurred in the overlapping heterogeneous DES group. In the overlapping DES-BMS group, the rate of MACE was 50% (three target-vessel revascularisations and one death after cardiogenic shock in a patient who developed ST-elevation MI 110 days after PCI).^22^

As stated in the results section, we used homogeneous drug-eluting stents in all 22 patients treated with overlapping DES, which matches the Burzotta study. However, we used a combination of DES and BMS in a much higher number of patients than in their registry (30 vs eight), and no MACE was experienced. In our study, MACE only included MI after 13.2 months in a patient treated with two overlapping Cyphers, yielding a MACE rate of 4.5%, compared to 0% in patients treated with DES-BMS. We must point out that we used the definition of a CKMB rise two-fold above baseline, although the new definition of MI is based on troponin level.^26^

Angiographic restenosis was detected in two of the six patients who had undergone follow-up angiography. Since we only performed follow-up angiography when considered clinically appropriate (in 11.5% of cases), the true angiographic restenosis rate in our cohort could not be established with certainty, compared to Burzotta *et al.*, who performed follow-up angiography in more than 80% of cases. In their experience, MACE was detected only in those patients who had undergone follow-up angiography and patients without follow-up angiography did not show MACE at nine months.

In the Burzotta study, total stent length was shorter in patients treated with DES-BMS than DES-DES [39 ± 16 mm in the DES-BMS vs 50 ± 10 mm in the overlapping Cypher and 42 ± 7 mm the overlapping Taxus group (*p* = 0.09)].^22^ Their results also showed more intimal hyperplasia at the site of stent overlap in DES-BMS overlaps than when homogeneous or heterogeneous DES were used. The higher late lumen loss translated into a higher in-segment binary restenosis rate in lesion segments covered with DES-BMS, therefore decreasing the possible benefits associated with DES implantation.

In the setting of long dissections, multiple short stent placements have proven to be equivalent to the use of long stents.^27^ In our practice, in four patients, all dissections were successfully treated with overlapping DES-BMS (Table 4). In addition, as stated in the results section, the additional bare-metal stents used for coverage of residual lesions, both in the case of dissections or for long lesions, were smaller and had larger diameters. Since the number of endpoints was small in our study, we may not reach a definite conclusion, but we hypothesise that the smaller lengths and larger diameters of the bare-metal stents used for coverage of residual lesions may have reduced the risk of MACE in the group treated with overlapping DES-BMS.

On the other hand, we found that lesions were estimated to be significantly shorter in the DES-BMS group, suggesting another potential factor for the reduced risk of MACE. However, total stent lengths were not significantly different between the two groups. This may be explained by the fact that all the patients treated with more than two stents were in the DES-BMS group, and additional bare-metal stents were used for the coverage of residual lesions.

Our results showed that bare-metal stents with relatively short lengths and large diameters can be overlapped in the proximal portion of a long drug-eluting stent for the coverage of residual lesions when the DES length would not suffice for the coverage of the total lesion, or in the case of proximal edge dissection. In our practice, this procedure was both feasible and safe, with no increased rate of late stent thrombosis, as opposed to the use of two or more drug-eluting stents, which have been frequently known to be associated with stent thrombosis, especially at the site of overlapping stent struts.

Limitations of this study include the fact that it was a retrospective analysis of consecutive and non-randomised patients, different stent types were used, and stent deployment techniques varied between the operators. Also, these data reflect the current clinical practice at our institution. Routine follow-up angiography was therefore not performed, which blurred the true rate of acquired restenosis within the study population.

## Conclusion

In clinical practice, there are patients in whom the interventional cardiologist is required to overlap two or more stents in order to cover residual lesions or dissecting flaps of a long atherosclerotic plaque. In our patients, there were no differences in mid-term clinical outcomes whether there was more than one overlapped drug-eluting stent, or a bare-metal stent had been deployed and overlapped in the proximal portion of a long drug-eluting stent.

Regarding the risk of late thrombosis when using two or more overlapped drug-eluting stents, our experience showed that this therapeutic strategy is both safe and feasible for the coverage of either dissections or residual lesions. Considering the small sample size of our study, a prospective, randomised clinical trial is recommended in future to determine the advantage of one technique over the other.
